# Qualitative evaluation of survey questions to assess treatment preference for daily oral or long‑acting injectable antiretroviral therapy among people living with HIV

**DOI:** 10.1371/journal.pone.0309588

**Published:** 2024-12-27

**Authors:** Cindy Garris, Irina Kolobova, Vasiliki Chounta, Alberto Díaz De Santiago, Robin Dretler, Tulika Singh, Gary Sinclair

**Affiliations:** 1 ViiV Healthcare, Durham, NC, United States of America; 2 ViiV Healthcare, Brentford, United Kingdom; 3 Infectious Diseases Division, Hospital Universitario Puerta de Hierro, Madrid, Spain; 4 Infectious Disease Specialists of Atlanta, Atlanta, GA, United States of America; 5 DAP Health, Palm Springs, California, United States of America; 6 PrismHealth North Texas, Dallas, Texas, United States of America; Beth Israel Deaconess Medical Center/Harvard Medical School, UNITED STATES OF AMERICA

## Abstract

Treatment of HIV has historically required taking daily oral antiretroviral therapy (ART). A recent alternative to daily oral ART is long-acting injectable ART with cabotegravir plus rilpivirine, administered monthly or every 2 months. The purpose of this qualitative study was to evaluate the concept relevance and interpretability of five previously developed questions: one treatment preference question and four questions designed to assess how the emotional burden associated with HIV treatment impacts treatment preferences. Thirty adults in the United States currently receiving HIV treatment were enrolled in a cross-sectional study involving one-on-one concept confirmation and cognitive debriefing interviews. Concept confirmation interviews included topics, questions, and probes designed to elicit information about the emotional burden of HIV and current perceptions of a participant’s treatment regimen. Cognitive debriefing assessed the relevance and clarity of instructions, questions, response options, and recall periods. Transcripts were analyzed with MAXQDA. Mean age of participants was 49 years (range: 29–68), with 60% being male and 40% female. Racial demographics included Blacks (40%), Whites (40%), and other (20%). During concept confirmation, participants endorsed concepts relevant to HIV treatment preference: fear of disclosure of HIV status (47%), forgetting to take daily oral medication (40%), and current treatment regimen as a bothersome daily reminder of HIV status (40%). During cognitive debriefing, participants interpreted the instructions, question, response options, and recall periods as intended for the treatment preference question. Additionally, participants confirmed that the preference question’s response options were appropriate and relevant to the experiences of people living with HIV. Participants also consistently interpreted the questions related to fear of disclosure of HIV status, anxiety associated with forgetting to take HIV medication, and HIV medication being an uncomfortable reminder of HIV status; however, participants provided variable responses to the question designed to assess treatment ease of use. These results support the concept relevance and interpretability of the single treatment preference question and three of the four emotional well-being questions among adults living with HIV.

## Introduction

An estimated 38 million people were living with HIV (PLHIV) at the end of 2021 [1]. Antiretroviral therapies (ART) have transformed HIV from a terminal illness to a chronic and manageable disease. ART has historically involved taking a combination of HIV medicines every day; however, some PLHIV experience a significant burden from taking daily oral ART. A daily oral regimen can be an uncomfortable reminder of their HIV status, the stigma associated with their disease, and the possibility that their HIV status might be accidentally disclosed. These challenges associated with living with HIV can influence regimen adherence and preference for alternative treatments that do not require daily oral administration [2–5]. Cabotegravir plus rilpivirine is the first complete long-acting, injectable ART regimen approved for HIV treatment in virologically suppressed adults and adolescents [6]. This regimen, approved by the Food and Drug Administration (FDA) in January 2021, is administered by a healthcare professional monthly or every two months, markedly reducing the frequency of ART administration to as few as six times a year. While some may prefer daily oral ART due to fewer clinic visits or avoidance of injections, a survey of 688 PLHIV found that 66% were interested in trying long-acting injectable ART, especially if daily oral ART resulted in unwanted medical or emotional consequences, such as gastrointestinal adverse events, suboptimal treatment adherence, privacy concerns, or emotional burden of daily dosing [3]. Similarly, a survey of 201 PLHIV found that most PLHIV would prefer injections every two months to a single daily oral tablet (59% vs 37%) [7]. A better understanding of preferences for daily oral or long-acting injectable ART would allow patients to be matched to the treatments that align with their preferences, values, and medical needs.

Patient preferences have been assessed in clinical trials comparing long-acting injectable ART with daily oral ART [8]. Established patient-reported outcome (PRO) instruments were used to assess satisfaction (HIVTSQ), acceptability of treatment (ACCEPT general domain), and tolerability of injections (PIN). Participants treated with long-acting injectable ART exhibited greater treatment satisfaction and acceptance than those treated with daily oral ART [8]. Among participants receiving long-acting injectable ART, the acceptability of injection site reactions significantly improved over time, and nearly all (≥97%) endorsed a preference for long-acting injectable ART as compared to their prior daily oral ART [8]. However, these instruments do not fully explore the complex psychosocial and preference factors that should inform the decision-making process for choice of daily oral or long-acting injectable ART.

In-depth interviews were conducted with participants in previous HIV clinical trials to gain a deeper understanding of the perceptions, experiences, and views of PLHIV regarding daily oral and long-acting injectable ART [9, 10]. Using thematic analysis of these qualitative interviews, we developed one question assessing patient preferences for long-acting injectable or daily oral ART and four questions assessing how the emotional burden facing PLHIV affects preferences for these treatment modalities. These emotional burden questions focused on concepts related to daily oral or long-acting injectable ART, including ease of use, fear of disclosure, adherence anxiety, and uncomfortable reminder of HIV status. In the ATLAS and FLAIR clinical trials, the single treatment preference question, which was administered to participants in the long-acting injectable ART arm only, asked participants if they preferred daily oral treatment or monthly injections [8]. Based on themes that emerged in qualitative interviews [9, 10], this question was revised to include multiple response options to assess the reasons for a person’s treatment preference and was used in the ATLAS-2M trial [11]. In addition, the revised treatment preference question and three of the four emotional well-being questions were used in the phase 3b SOLAR trial [12] and other real-world studies [13, 14]. However, the conceptual relevance of these questions has only been evaluated in a clinical trial population, limiting the generalizability of clinical trials results to a broader HIV population [9, 10]. Moreover, the clarity and interpretability of these questions have not been established.

The Food and Drug Administration (FDA) guidance recommends that patient feedback is critical to establishing the content validity of PRO instruments [15, 16]. The purpose of this study was to confirm whether the concepts included in the questions are relevant in a general HIV population and to evaluate individuals’ understanding of the instructions, questions, response options, and recall period. By establishing the relevance and interpretability of these questions, they can be used in research studies of ART treatment modalities to complement clinical outcomes and help health care professionals better understand patient treatment preferences and the emotional impact of treatment regimens on the daily lives of people with HIV.

## Materials and methods

### Survey questions

**Fig 1:**
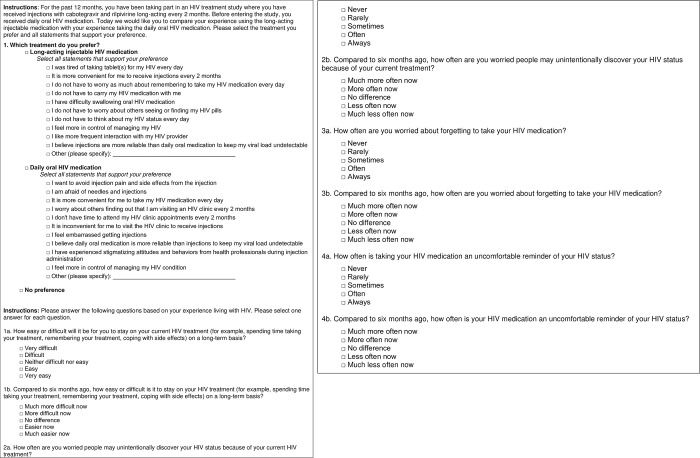
Original survey assessing treatment preference and emotional well-being.

To assess the relevance and interpretability of the survey questions, a cross-sectional qualitative study was conducted that included concept confirmation and cognitive debriefing interviews. A treatment preference question and four emotional well-being questions were included in this assessment (**Fig 1**). The treatment preference question was developed to assess preference for daily oral ART or long-acting injectable ART. This question has several response options that allow respondents to indicate their preference based on various factors, including convenience, concerns about medication administration, impact on daily life, perception of reliability in maintaining viral load, interaction with healthcare providers, and personal experiences. In addition to the preference question, four single-item questions were developed to assess additional factors that contribute to preferences for daily oral ART or long-acting injectable ART. These questions address four common concepts experienced by PLHIV, including ease of use, fear of disclosure, adherence anxiety, and treatment-related daily reminder of HIV status. Each of the four questions originally had two versions with different recall periods (immediate and 6 months); however, a third recall period at 12 months was added during cognitive debriefing interviews. These recall periods align with commonly used timepoints for safety and efficacy assessments in HIV trials and FDA guidance [17]. By measuring patient responses at baseline, 6 months, and 12 months, emotional changes in patients after exposure to treatment can be assessed over time and compared to changes in clinical outcomes. Unlike the preference question, which was designed to be used in persons receiving long-acting injectable ART, these four questions were developed to assess factors that influence treatment preference among those receiving either daily oral ART or long-acting injectable ART.

### Recruitment procedures

Potentially eligible participants were identified through an existing patient database and referral network within the US. Eligible participants included those who were 18 years or older and currently receiving treatment for HIV. Participants were required to have adequate English language skills and sufficient cognitive ability to participate in a qualitative interview. Participants were also required to complete a screening and demographic form. All participants provided written informed consent prior to participating in the interview and were compensated $75 after the interview was completed. The study was approved by the New England Institutional Review Board (IRB) prior to contacting participants. A target sample of 30 participants was the goal for confirming the relevance and interpretability of the questions. All interviews were completed between May 4, 2020, and May 21, 2020.

### Concept confirmation and cognitive debriefing

**Fig 2:**
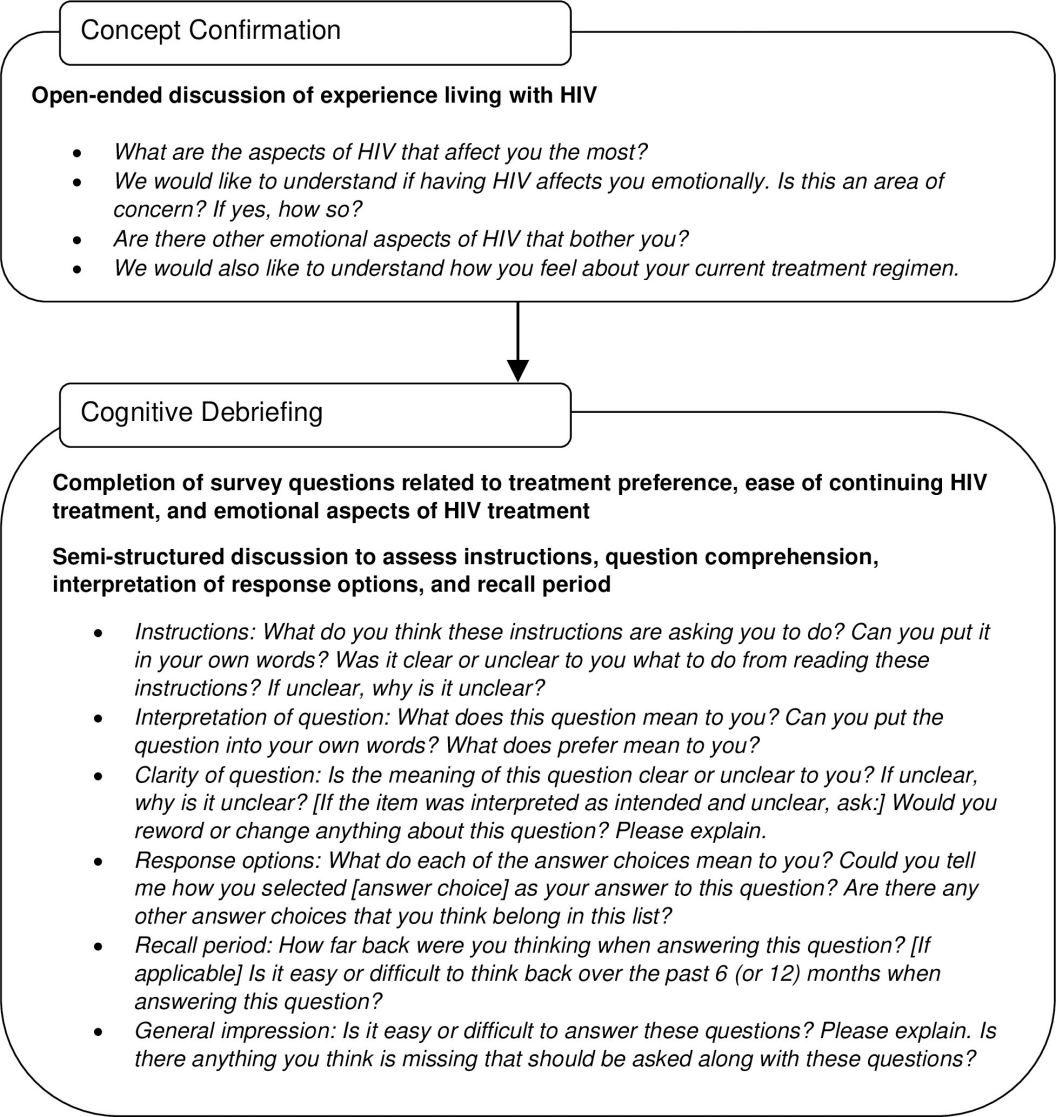
Interview summary.

All interviews were conducted using a semi-structured interview guide with an initial concept confirmation discussion followed by a cognitive debriefing interview (**Fig 2**).

The concept confirmation portion of the interview was conducted to elicit spontaneous descriptions from participants and confirm the conceptual coverage of the questions that emerged in previous qualitative interviews [9, 10]. The interview guide included topics, questions, and probes designed to elicit information about the emotional burden of HIV and current perceptions of the participant’s treatment regimen. Participants were then engaged in a cognitive debriefing discussion following best practices guidelines for developing PROs [15, 18]. The goal of the cognitive debriefing discussion was to evaluate the participants’ interpretation of the instructions and HIV questions and to ensure the response options and recall periods were appropriate for capturing the experiences of PLHIV. Participants were also asked about the comprehensiveness, format, and relevance of the HIV question content. The format of the cognitive debriefing interview involved a think-aloud approach, in which participants verbalized their thoughts while responding to each question. If a participant’s verbal response was incomplete, the interviewer asked specific questions to prompt the participant to address the interpretation, clarity, and ease of completion of the question or responses; comprehensiveness of the response options; appropriateness of the format, response scales, and recall periods.

To assess whether participants used each response option in the treatment preference question, participants were asked to complete the question while imagining themselves in two hypothetical circumstances: one where they preferred long-acting injectable HIV medication and one where they preferred daily oral HIV medication.

All interviews were conducted virtually on a web-based platform to allow participants to view the survey questions. Interviews were conducted by interviewers trained in qualitative interviewing methodology. All interviews were conducted in English, took 30–45 minutes to complete, and were audio-recorded and subsequently transcribed. The total sample (n = 30) was divided into three interview cohorts to allow for interim data analysis of content validity after each cohort. Fourteen participants were interviewed in the first cohort, followed by a second cohort of 12 participants and a final confirmatory cohort of 4 participants. Depending on participant feedback, questions were revised if necessary and retested in the subsequent cohort.

### Data analysis

The coding process was guided by established qualitative research methods, including grounded theory and constant comparative method [19, 20]. Specific grounded theory methods that were applied included: letting the coding scheme be dictated by the data and not preconceived notions, constantly comparing and contrasting concepts to inform relationships among the data (i.e., constant comparative method) and leaving comments to explain findings and inform the next step of analysis (i.e., harmonizing codes). A coding scheme, initially developed based on the discussion guide and research objectives, was updated as necessary to incorporate newly emerging data based on participant responses. Codes were applied to text characterizing a concept within each transcript and then queried for frequency across transcripts. Unique concepts were identified and ultimately formed broader categories that helped to identify and explain patterns and relationships within the data [21, 22]. Frequencies of unique concepts were reported with accompanying exemplary quotes. Interview transcripts were analyzed with content analysis software (MAXQDA version 18.2.; Berlin, Germany). Throughout the coding process, the frequency of themes emerging from the transcripts was monitored to ensure the thematic relevance of the primary concepts of the survey questions. Demographic characteristics and health information of the participants were summarized and reported as frequencies.

## Results

### Demographics and clinical characteristics

Thirty participants were interviewed during May 2020, prior to commercial availability of long-acting injectable ART with cabotegravir plus rilpivirine (**Table 1**). Participants were recruited from geographically diverse regions within the United States, with most located in Texas (n = 8, 27%), New York (n = 6, 20%), and California (n = 3, 10%). The mean age of participants was 48.5 years. Most participants were male (n = 18, 60%) and homosexual (n = 17, 56%). Equal numbers of Whites (n = 12, 40%) and Blacks (n = 12, 40%) were enrolled. Two thirds of the sample (67%) had at least some college education.

**Table 1:** Demographic and clinical characteristics.

Characteristic	N = 30
**Age (years)**	
Mean	48.5
Range	29–68
**Sex, n (%)**	
Male	18 (60)
Female	12 (40)
**Sexual orientation, n (%)**	
Homosexual (gay/lesbian)	17 (57)
Heterosexual/straight	10 (33)
Bisexual	3 (10)
**Race/ethnicity, n (%)**	
White, non-Hispanic	12 (40)
Black/African American	12 (40)
Hispanic/Latinx	6 (20)
**Education status, n (%)**	
Some high school	1 (3)
High school degree	9 (30)
Some college	12 (40)
College degree	6 (20)
Postgraduate degree	2 (7)
**Location, n (%)**	
Texas	8 (27)
New York	6 (20)
California	3 (10)
Georgia	2 (7)
Massachusetts	2 (7)
Other^a^	9 (30)
**Time since HIV diagnosis, n (%)**	
2–5 years	8 (27)
6–10 years	10 (33)
11–15 years	6 (20)
15+ years	6 (20)
**Level of HIV impact on daily life, n (%)**	
Not at all	3 (10)
A little	10 (33)
A moderate amount	9 (30)
A significant amount	6 (20)
It overpowers my life	2 (7)

^a^One patient was from each of the following 9 states: Connecticut, Florida, Illinois, Kentucky, Maryland, New Jersey, Pennsylvania, Washington, and Washington DC.

Clinical characteristics of participants are displayed in **Table 1**. Most participants had been living with HIV for less than 10 years (n = 18, 60.0%). Nearly all participants reported that HIV had an impact on their daily life: 19 (63.3%) participants reported that HIV had “a little” or “a moderate” impact on their daily life, while 8 (26.7%) reported that it had “a significant” or “overpowering” impact on their daily life. All participants were currently receiving daily oral treatment for HIV. The most common daily oral regimens were Genvoya® (elvitegravir, cobicistat, emtricitabine, and tenofovir alafenamide; 33.3%), Biktarvy® (bictegravir, emtricitabine, and tenofovir alafenamide; 26.7%), and Triumeq® (dolutegravir, abacavir, and lamivudine; 13.3%). None of the participants were currently or had previously received treatment with cabotegravir plus rilpivirine long-acting regimen.

### Concept confirmation

Participants described many ways that HIV affects their daily lives (**S1 Table**). Commonly reported experiences among PLHIV included sadness/depression (n = 19, 63.3%), fear of disclosure of their HIV status (n = 14, 46.7%), and feeling isolated or lonely (n = 13, 43.3%). Participants also expressed concerns with barriers to medication access (n = 16, 53.3%) and forgetting to take their daily oral medication (n = 12, 40.0%). Most participants (n = 22, 73.3%) highlighted that their current oral treatment regimen resulted in a daily reminder of their HIV status, and 12 (40.0%) reported that this reminder was bothersome. Despite the daily reminder, most participants (n = 24, 80.0%) found their current treatment regimen easy to manage; however, some participants (n = 6, 20.0%) found their current HIV treatment difficult. Moreover, when participants were asked about ways to improve long-term adherence to treatment, most endorsed a new mode of administration (n = 17, 56.7%), less frequent dosing (n = 16, 53.3%), and reduced cost (n = 10, 33.3%). Participant also reported concerns related to being ashamed or embarrassed, which is a concept related to fear of disclosure. Other concepts identified by at least 20% of participants included “More support” or “Reduced cost.” Both concepts were considered related to social support, disease status, insurance coverage, personal finances, or healthcare policy rather than treatment. Importantly, the commonly reported experiences of PLHIV were relevant to concepts in the treatment preference question and emotional well-being questions.

### Cognitive debriefing

#### Treatment preference question

**Fig 3:**
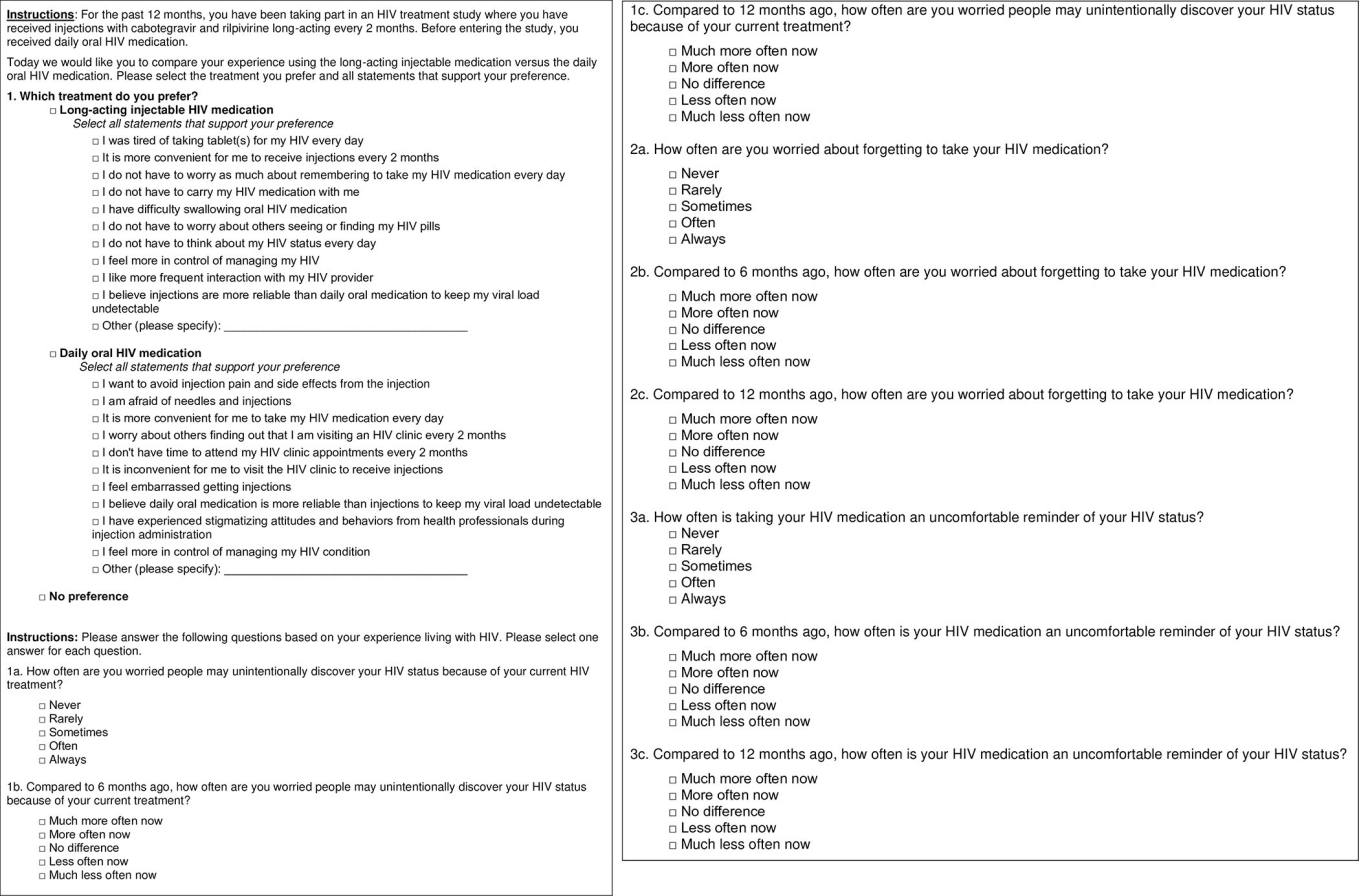
Revised survey assessing treatment preference and emotional well-being.

Participant feedback on the treatment preference question was positive and led to only minor modifications to the instructions. All participants from cohort 1 (n = 14 of 14, 100.0%) interpreted the instructions as intended; however, four participants (n = 4 of 14, 28.6%) noted that the instructions were too lengthy and recommended revising the instructions to make them easier to read. One participant recommended replacing the text “with your experience taking” with “versus” in the instructions and dividing the instructions into two separate paragraphs to make them easier to read. The revised instructions were clear and acceptable to the remaining participants from cohorts 2 and 3 (n = 16, 100.0%), and no further changes were made (**Fig 3**). All participants (n = 30, 100.0%) interpreted the question and response options as intended.

To evaluate the relevance of the response options, all 30 participants were asked to complete the treatment preference question by first imagining that they preferred long-acting injectable HIV medication and then to imagine they preferred daily oral HIV medication. All response options were selected at least once, and participants were allowed to select all options that might apply. One participant selected “other” as an option but did not specify an additional response option.

Participants were asked to describe why they selected a response option if they preferred long-acting injectable HIV medication (**S2 Table**). Convenience emerged as a recurring theme, with participants expressing a desire for less frequent dosing and the elimination of concerns about remembering to take medication daily. One participant reported their reason for preferring injections every 2 months as follows:

*“Absolutely, I agree that would be pretty convenient. It would remove a step for me at night and also, I, if I were to have anxiety about missing, it would eliminate that completely. So, I think that’s positive.”*

The appeal of not having to carry HIV medication and the potential to avoid others seeing or finding their pills were also mentioned. Difficulties with swallowing oral medication, the perceived reliability of injections, and the importance of open communication and frequent interaction with healthcare providers were additional factors influencing preferences. Participants also highlighted the sense of control and freedom from daily worry that long-acting injectable ART could provide.

Participants were also asked to describe why they selected a response option if they preferred daily oral HIV medication (**S2 Table**). Participants expressed apprehensions about injection-related pain and potential side effects, while others mentioned a fear of needles and injections. Convenience and adherence to daily routines were valued by some participants who preferred the simplicity of taking medication orally. Concerns about privacy and the fear of others discovering their HIV status were also highlighted. Inconvenience related to work schedules and personal life were also mentioned as a factor influencing preferences. Embarrassment regarding injections and the potential exposure of intimate areas was a concern for some participants. Familiarity and comfort with oral medication were mentioned by others. Participants also reported their concern with stigmatizing attitudes from healthcare professionals during injection administration. Finally, the desire for personal control and self-reliance was emphasized, suggesting that oral medication may provide a sense of empowerment to some PLHIV. One participant explained their reason for preferring daily oral medication as follows:

*“And I will say this statement does apply because people want to feel more in control when they do things themselves, and don’t have to necessarily rely on someone else, or something else. And having oral medication gives them that opportunity to be in control.”*

Taken together, these data demonstrate that participants utilize all response options in the treatment preference question and could easily describe how they arrived at their answers.

#### Survey questions related to the emotional burden associated with HIV treatment

Participants were asked four questions related to the emotional burden associated with HIV treatment. All participants (n = 30 of 30, 100.0%) interpreted the instructions, response options, and most questions as intended. However, the question on the ease or difficulty of continuing HIV treatment on a long-term basis was extensively revised (**S1 Fig**). Some participants in cohort 1 found this question wordy (n = 3, 21.4%) or difficult to comprehend (n = 5, 35.7%). As a result, the language was simplified and examples added to clarify the intended meaning of the question. Participants in cohort 2 continued to have difficulty interpreting the question correctly, and the wording was again modified. After these revisions, the 4 participants in cohort 3 interpreted the question as intended; nevertheless, given the difficulty that most participants had interpreting this question and the small size of cohort 3, the question was deemed not to have been proven to be a valid measure to assess the ease or difficulty of long-term adherence to HIV treatment.

No changes were adopted for the remaining questions related to the emotional burden of HIV treatment (**Fig 3**). All participants (n = 30 of 30, 100.0%) interpreted these questions and their response options as intended. For the core question related to fear of disclosure of HIV status, one participant recommended adding new response options (“almost never” and “almost always”); this suggestion was pilot tested in cohort 2, but most participants (n = 10 of 12, 83.3%) preferred the simpler scale. This change was not adopted in the final question in cohort 3. Participants were also asked if “worried” was an appropriate term, and most (n = 25 of 30, 83.3%) found it appropriate. Participants were also asked to comment on the appropriateness of the term “uncomfortable reminder,” and again most (n = 26 of 30, 86.7%) found it appropriate. Those individuals who did not like the terms did not suggest alternative wording.

Each emotional well-being question had an immediate, 6-month, and 12-month recall period. Participants reported that all recall periods and response options were acceptable. A few participants in cohort 1 (n = 3 of 14, 21.4%) reported that the question was wordy and recommended revisions; a minor edit to simplify the recall question was made (i.e., “compared to six months ago” was changed to “compared to 6 months ago”). A 12-month recall period was added in interviews with cohorts 2 and 3. All participants in cohorts 2 and 3 (n = 16 of 16, 100.0%) reported that they would be able to accurately recall changes in the previous 12 months. The reason that it is easy to recall treatment-related events among PLHIV was illustrated by one participant:

*“It’s easy. With a chronic condition like this, you remember how things are and if they have stayed the same or if they have changed. It’s not like you are asking me my coffee order from a year ago. This is something that I do regularly that is on my mind. I’d be able to remember if there was a change over time.”*

The relevance of the response options for the emotional burden questions with immediate, 6-month, and 12-month recall periods was evaluated. Participants were asked to complete the questions based on their own experience with HIV treatment. Overall, participants used nearly all response options, except for a few response options for questions with 6-month and 12-month recall periods. This finding may indicate that participant attitudes and experience with HIV medication were generally stable during the previous 6 and 12 months.

## Discussion

Thirty PLHIV receiving daily oral ART completed concept confirmation and cognitive debriefing interviews to assess treatment preference and the emotional burden associated with HIV treatment. Results from the semi-structured, in-depth interviews confirmed that fear of disclosure, adherence anxiety, treatment convenience, and treatment-related daily reminder of HIV status are relevant to PLHIV. These results are consistent with the results of previous qualitative studies [9, 10]. Previous interviews with PLHIV in other studies showed that participants preferred many aspects of long-acting injectable ART to daily oral ART, including the convenience of less frequent dosing and the emotional benefits of reduced fear of accidental HIV disclosure, removal of the daily reminder of HIV status, and reduced stigma associated with carrying or taking pills. In the present concept confirmation study, participants also endorsed fear of disclosure of their HIV status, forgetting to take their daily oral medication, and their current treatment regimen being a daily reminder of their HIV status. Although most participants found their current treatment regimen easy to manage, all participants in this study were currently receiving daily oral ART and had not received long-acting injectable ART. Most participants, however, endorsed a new mode of treatment administration with less frequent dosing. Cognitive debriefing was used to further assess the questions’ relevance and comprehensibility. Cognitive debriefing with participants demonstrated that the questions and response options contain concepts that are relevant to the experiences of PLHIV and confirmed that no concepts were missing. Participant feedback demonstrated that the questions related to treatment preference, fear of disclosure, adherence anxiety, and uncomfortable reminder of HIV status were interpreted as clear, comprehensive, and relevant to the experiences of PLHIV. These findings establish the content validity of the treatment preference question and three of the four tested questions on the emotional impact of HIV treatment in a non-clinical trial HIV population. The question related to the ease or difficulty of long-term adherence to HIV treatment was not interpreted in the same way by participants; thus, its content validity was not clearly demonstrated, and this question was excluded from the final version.

For each single-item question on the challenges related to HIV treatment, a new item was added with an extended recall period of the past 12 months. The purpose of this recall period was to test the appropriateness of asking participants to think back to their state one year before. Qualitative data collected on these recall periods support a participant’s ability to recall both the past 6 and 12 months with relative ease. This finding is consistent with literature supporting the appropriateness of an extended recall period for salient life events, such as an HIV diagnosis and the challenges of managing this chronic illness [23, 24]. Additionally, PRO guidelines support the use of a recall period that corresponds to patient characteristics and overall study design [25]. Many clinical trials of ART assess outcome measures using a 12-month follow-up period, thus making these questions with 6- and 12-month recall periods appropriate tools for assessing PROs in HIV clinical trials.

During in-depth interviews, many participants spontaneously reported a desire for new non-oral HIV treatment administration options before learning that the questions were related to a long-acting injectable treatment option. This finding is consistent with the discussion topics from the Patient-Focused Drug Development for HIV public meeting hosted by the FDA in 2013 [26]. A key theme to emerge from this discussion was that individuals, while acknowledging advances in HIV therapy, desire better treatments that remove the need for daily adherence. In the 2019 Positive Perspectives Study, 55% of respondents expressed openness towards longer-acting (non-daily) HIV medication [4]. Moreover, 33% of respondents indicated that having to remember to take their HIV medication every day causes them stress and anxiety [5]. Respondents were also asked to prioritize potential improvements to HIV medicines. The most important improvements were reducing the long-term bodily effects of treatment (47%), followed by having longer-lasting treatment options (43%) with fewer side effects (41%) and having less HIV medicine each day as long as it was equally effective (25%) [4]. Additionally, previous qualitative studies demonstrated that many PLHIV would prefer to receive long-acting injectable ART [9, 10]. Participants interviewed in these prior qualitative studies reported preferring long-acting injectable ART to daily oral pills because it was more convenient and had more emotional benefits, including a reduced risk of HIV disclosure and fewer daily reminders of HIV status. In the ATLAS and FLAIR clinical trials, 86–91% of participants reported preferring long-acting injectable ART to daily oral ART after 48 weeks of treatment [8]. Taken together, these previous studies support the conclusion that many in the HIV community desire to have alternative treatment options.

The HIV questions validated in this study have been used in clinical trials assessing the efficacy and safety of daily oral or long-acting injectable ART regimens [12, 27]. The treatment preference question has been used in its revised form (**Fig 3**) in the ATLAS-2M and SOLAR trials, and three of the four emotional well-being questions have been used in their revised form (**Fig 3**) in the SOLAR trial [27] and observational real-world studies [14, 26]. Results from these studies will be important for assessing preference and reasons for preference for daily oral or long-acting injectable ART.

A strength of the current study was that it included 30 participants from geographically diverse regions of the United States and with diverse sexual orientations, racial identities, and educational levels. Another strength of this research is that it has successfully demonstrated that cognitive debriefing research can be conducted remotely using a web-based browser to visualize questions. One limitation of this study is that it lacked information on other clinical characteristics or psychosocial constructs that might influence participant feedback or HIV treatment experience. Additionally, other factors besides treatment attributes can influence ART treatment choices (e.g., costs, insurance coverage, lack of approvals, pregnancy, and scale-up), and we identified two non-treatment-related concepts (“More support” or “Reduced cost”) that were not represented in the survey but were reported by at least 20% of participants. These concepts could be explored in real-world studies of HIV treatments. Finally, despite being conceptually validated only in the English language in a sample selected from a well-educated US population, the questions were translated into multiple languages for use in the SOLAR trial [12].

Although this study aimed to confirm that concepts included in the survey were patient relevant, it did not aim to establish that all relevant concepts were identified. While previous studies focused on concepts related to the benefits and emotional burden of long-acting injectable versus daily oral ART in participants in a clinical trial [9, 10], our concept confirmation study explored the relevance of these concepts in a population more representative of the general HIV population. Participants were asked open-ended questions to encourage robust qualitative data, but they were also asked more structured questions to evaluate the conceptual coverage, interpretability, and clarity of the draft survey. The concept confirmation portion of the interviews included topics, questions, and probes designed to elicit information about the emotional burden of HIV and current perceptions of a participant’s treatment regimen. Although codes were applied to text characterizing a concept within each transcript and then queried for frequency across transcripts, a full saturation analysis was not conducted. Instead, we assessed the frequency of survey-related concepts to determine their relevance to a representative population of people living with HIV.

## Conclusions

In summary, the present qualitative study supports the content validity of the treatment preference question and three of the four questions related to the emotional impact of HIV treatment. These questions were well understood by PLHIV and reflected concepts appropriate to their experiences with HIV treatment. The validated form of these questions have been used in both randomized clinical trials and real-world studies to assess treatment satisfaction and emotional well-being [12, 13, 28]. Findings from these and future studies hold significant potential to enhance understanding of how the attributes of long-acting injectable and daily oral medications influence patient preferences.

## Supporting information

S1 Table Emotional burden concepts related to the experiences of people living with HIV. (DOCX)

S2 Table Participant quotes related to responses selected for long-acting injectable HIV medication or daily oral HIV medication. (DOCX)

S1 Fig Original and revised questions related to the ease or difficulty of long-term HIV treatment. (TIF)

## References

[1] World Health Organization. HIV. 2022.

[2] Clark L, Karki C, Noone J, Scherzer J, Bode M, Rizzini P, et al. Quantifying people living with HIV who would benefit from an alternative to daily oral therapy: perspectives from HIV physicians and people living with HIV. Population Medicine. 2020;2(October):1–18.

[3] Akinwunmi B, Buchenberger D, Scherzer J, Bode M, Rizzini P, Vecchio F, et al. Factors associated with interest in a long-acting HIV regimen: perspectives of people living with HIV and healthcare providers in four European countries. Sex Transm Infect. 2021;97(8):566–73. Epub 2021/02/27. doi: 10.1136/sextrans-2020-054648 . 33632889

[4] de los Rios P, Okoli C, Young B, Allan B, Castellanos E, Brough G, et al. Treatment aspirations and attitudes towards innovative medications among people living with HIV in 25 countries. Population Medicine. 2020;2(July). doi: 10.18332/popmed/124781

[5] de Los Rios P, Okoli C, Castellanos E, Allan B, Young B, Brough G, et al. Physical, Emotional, and Psychosocial Challenges Associated with Daily Dosing of HIV Medications and Their Impact on Indicators of Quality of Life: Findings from the Positive Perspectives Study. AIDS Behav. 2021;25(3):961–72. Epub 2020/10/08. doi: 10.1007/s10461-020-03055-1 ; PubMed Central PMCID: PMC7936969. 33026574 PMC7936969

[6] Food and Drug Administration. CABENUVA (cabotegravir extended-release injectable suspension; rilpivirine extended-release injectable suspension) Prescribing Information. 2022.

[7] Matza LS, Howell TA, Chounta V, van de Velde N. Patient preferences and health state utilities associated with the treatment process of antiretroviral therapy for people living with HIV. Qual Life Res. 2023;32(2):531–41. Epub 2022/12/14. doi: 10.1007/s11136-022-03290-0 ; PubMed Central PMCID: PMC9746581. 36512302 PMC9746581

[8] Murray M, Antela A, Mills A, Huang J, Jäger H, Bernal E, et al. Patient-Reported Outcomes in ATLAS and FLAIR Participants on Long-Acting Regimens of Cabotegravir and Rilpivirine Over 48 Weeks. AIDS Behav. 2020;24(12):3533–44. Epub 2020/05/25. doi: 10.1007/s10461-020-02929-8 ; PubMed Central PMCID: PMC7667137. 32447500 PMC7667137

[9] Kerrigan D, Mantsios A, Gorgolas M, Montes ML, Pulido F, Brinson C, et al. Experiences with long acting injectable ART: A qualitative study among PLHIV participating in a Phase II study of cabotegravir + rilpivirine (LATTE-2) in the United States and Spain. PLoS One. 2018;13(1):e0190487. Epub 2018/01/06. doi: 10.1371/journal.pone.0190487 . 29304154 PMC5755771

[10] Mantsios A, Murray M, Karver TS, Davis W, Margolis D, Kumar P, et al. Efficacy and Freedom: Patient Experiences with the Transition from Daily Oral to Long-Acting Injectable Antiretroviral Therapy to Treat HIV in the Context of Phase 3 Trials. AIDS Behav. 2020;24(12):3473–81. Epub 2020/05/16. doi: 10.1007/s10461-020-02918-x . 32410051

[11] Chounta V, Overton ET, Mills A, Swindells S, Benn PD, Vanveggel S, et al. Patient-Reported Outcomes Through 1 Year of an HIV-1 Clinical Trial Evaluating Long-Acting Cabotegravir and Rilpivirine Administered Every 4 or 8 Weeks (ATLAS-2M). The Patient—Patient-Centered Outcomes Research. 2021;14(6):849–62. doi: 10.1007/s40271-021-00524-0 34056699 PMC8563641

[12] Ramgopal MN, Castagna A, Cazanave C, Diaz-Brito V, Dretler R, Oka S, et al. Efficacy, safety, and tolerability of switching to long-acting cabotegravir plus rilpivirine versus continuing fixed-dose bictegravir, emtricitabine, and tenofovir alafenamide in virologically suppressed adults with HIV, 12-month results (SOLAR): a randomised, open-label, phase 3b, non-inferiority trial. Lancet HIV. 2023. Epub 2023/08/12. doi: 10.1016/s2352-3018(23)00136-4 . 37567205

[13] Scherzer J NS, Jonsson- Oldenbüttel C, Wyen C, Esser S, Weinberg G, Potthoff A, Krznaric I, et al. Perceptions of cabotegravir + rilpivirine long-acting (CAB+RPV LA) from people living with HIV (PLHIV) in the CARLOS study. 12th IAS Conference on HIV Science; July 23–26, 2023; Brisbane, Australia2023. p. 686.

[14] Valenti W, Dandachi D, Cunningham D, Hsu R, Nguyen K, Teichner P, et al., editors. Perspectives of People With HIV 12 Months Following a Switch to Cabotegravir and Rilpivirine Long-Acting (CAB+RPV LA) in an Observational Real-world US Study (BEYOND). 25th International AIDS Conference; 2024 July 22–26; Munich, Germany.

[15] Food and Drug Administration. Guidance for Industry Patient-Reported Outcome Measures: Use in Medical Product Development to Support Labeling Claims. 2009. p. 1–35. 10.1186/1477-7525-4-79 PMC1629006 17034633

[16] Food and Drug Administration. Patient-Focused Drug Development: Collecting Comprehensive and Representative Input Guidance for Industry, Food and Drug Administration Staff, and Other Stakeholders [Draft guidance]. 2018.

[17] Food and Drug Administration. Human Immunodeficiency Virus-1 Infection: Developing Antiretroviral Drugs for Treatment Guidance for Industry. 2015.

[18] Food and Drug Administration. Patient-Focused Drug Development: Methods to Identify What Is Important to Patients Guidance for Industry, Food and Drug Administration Staff, and Other Stakeholders [Draft guidance]. 2019.

[19] Charmaz K, Smith J, Harré R, Van Langenhove L. Grounded Theory. Rethinking methods in psychology. London: Sage Publications; 1995. p. 27–49.

[20] Glaser B, Strauss A. The Discovery of Grounded Theory: Strategies for Qualitative Research. The Discovery of Grounded Theory. New York: Aldine de Gruyter; 1967. p. 1–18.

[21] Glaser BG, Strauss AL. The constant comparative method of qualitative analysis. The Discovery of Grounded Theory: Strategies for Qualitative Research. New York: Aldine de Gruyter; 1967. p. 101–15.

[22] Lasch KE, Marquis P, Vigneux M, Abetz L, Arnould B, Bayliss M, et al. PRO development: Rigorous qualitative research as the crucial foundation. Quality of Life Research. 2010;19:1087–96. doi: 10.1007/s11136-010-9677-6 . 20512662 PMC2940042

[23] Kjellsson G, Clarke P, Gerdtham U-G. Forgetting to remember or remembering to forget: A study of the recall period length in health care survey questions. Journal of Health Economics. 2014;35:34–46. doi: 10.1016/j.jhealeco.2014.01.007 24595066

[24] Statistics VaH. Series 6, Cognition and Survey Measurement. US Dept of Health and Human Services, Public Health Service, Centers for Disease Control, National Center for Health Statistics. 1989.

[25] Norquist JM, Girman C, Fehnel S, DeMuro-Mercon C, Santanello N. Choice of recall period for patient-reported outcome (PRO) measures: criteria for consideration. Qual Life Res. 2012;21(6):1013–20. Epub 2011/09/13. doi: 10.1007/s11136-011-0003-8 . 21909804

[26] Food and Drug Administration. The Voice of the Patient: HIV Patient-Focused Drug Development and HIV Cure Research. 2014.

[27] Overton ET, Richmond G, Rizzardini G, Thalme A, Girard PM, Wong A, et al. Long-Acting Cabotegravir and Rilpivirine Dosed Every 2 Months in Adults With Human Immunodeficiency Virus 1 Type 1 Infection: 152-Week Results From ATLAS-2M, a Randomized, Open-Label, Phase 3b, Noninferiority Study. Clin Infect Dis. 2023;76(9):1646–54. doi: 10.1093/cid/ciad020 ; PubMed Central PMCID: PMC10156123. 36660819 PMC10156123

[28] Chounta V MC, Cazanave C, Adachi E, Eu B, Montero Alonso M, Crofoot G, et al. Patient-reported outcomes after 12 months of maintenance therapy with cabotegravir + rilpivirine long-acting compared with bictegravir/emtricitabine/tenofovir alafenamide in the Phase 3b SOLAR study. 12th IAS Conference on HIV Science; July 23–26, 2023; Brisbane, Australia2023. p. 98–9. 10.1007/s10461-024-04490-0 PMC11739242 39375290

