# Supplementary material for: Qualitative evaluation of survey questions to assess treatment preference for daily oral or long‑acting injectable antiretroviral therapy among people living with HIV
Source: PLoS One. 2024 Dec 27;19(12):e0309588. doi: 10.1371/journal.pone.0309588 (PMC11676866; doi:10.1371/journal.pone.0309588)
Supplement: S1 Table — (DOCX) [file pone.0309588.s001.docx]

| **Supplementary Table S1:** Emotional burden concepts related to the experiences of people living with HIV | | |
| --- | --- | --- |
| **Concept** | **n (%)** | **Representative quotes (Participant ID)** |
| **Emotional impact** |  | |
| Sadness/depression | 19 (63.3) | *“I’m really emotional. I just think for me, it’s just very emotional thing. Like every time I take [my medication] I’m just getting more and more depressed throughout like everything. I just don’t want to think about it.” (25)* |
| Fear of disclosure | 14 (46.7) | *“I was scared that people would find out my status, either by like someone telling someone else, or someone snooping around my house and finding the bottles or even going through my trash – I know that sounds crazy, but I was just so scared that someone would violate my privacy.” (05)* |
| Isolation/lonely | 13 (43.3) | *“There are definitely times where I feel like nobody really understands what it feels like, the struggles in my mind” (01)* |
| Ashamed / embarrassed | 7 (23.3) | *“It’s very, very embarrassing …Because I became positive from my significant other, and it’s degrading because when I admit that I’m positive they think I’m an IV user. I’m not an IV user. They think that I was a street walker. And I wasn’t a street walker. …I’m very ashamed of being HIV positive” (17)* |
| Self-conscious | 3 (10.0) | *“It’s kind of like self-esteem and whatnot. It’s like just because there’s such a stigma on it … it does a lot to my self-esteem. (34)* |
| Angry | 2 (6.7) | *“[I feel] angry and sometimes hostile …it does cause my mood to shift often because this is just unfortunate, but it’s something that I have to learn how to cope with” (29)* |
| **Adherence and compliance** |  | |
| Access barriers | 16 (53.3) | *“The only worry I would face as far as my treatment is the ability to get the medication. I do qualify for programs that help me cover the cost, but … I haven’t actually been able to go to any clinic, yet. Luckily, I did have kind of a small stockpile of medications for myself, but I guess so the access to my medication would be more so my only fears” (09)* |
| Forgetfulness | 12 (40.0) | *“It’s still a struggle. It’s something that I should remember and there’s times that I don’t and when my partner remembers, but not me. So, he’s reminding me now [to take my medication]” (12)* |
| **Daily reminder of HIV** |  | |
| Bothersome | 12 (40.0) | *“The medicine does play off a reminder that I do have HIV, and I did make a mistake somewhere in my life, and it really just causes me to feel a lot of guilt and remorse.” (29)* |
| Not bothersome | 10 (33.3) | *“It doesn’t bother me a bit because me having HIV doesn’t -- I have no problem with it. I’ve had it so long it doesn’t bother me a bit” (03)* |
| No daily reminder | 8 (26.7) | *“I would say that I don’t think of it as a daily reminder” (31)* |
| **Current treatment** |  | |
| Easy | 24 (80.0) | *“It can’t get no easier than what it is to say taking one pill, you know, because -- oh, and the fact that the pill is smaller, because I had an issue with my other pills, they were like so big … Now, this last medication I’m on, it works so much better. The pill is not big. I’m able to swallow it without any issues, you know, and like I said, it works” (07)* |
| Difficult | 6 (20.0) | *“The worst thing about HIV are the side effects of the treatment. I know they are a lot better than they used to be, but still, I desperately want a treatment that isn’t hard on my body on a daily basis to take. If they had something that didn’t upset my stomach or make me feel weak, that would be wonderful” (15)* |
| **Improving long-term treatment** |  | |
| Different mode of administration | 17 (56.7) | *“I’ve read studies about being able to utilize more like the birth control like things where you could get, like those -- the birth control where they can put it in your arm or whatever, and it’s there for two or three months, and you don’t have to take it every day. Now I think they are also experimenting with injectable that you just had to get injected once a month with something that would keep you undetectable for over a longer period of time” (20)* |
| Less frequent dosing | 16 (53.3) | *“It would be easier if you could take one pill a month, or once a week that would make it way easier. …Because you don’t have to think about it. I mean, and again because we do have to take meds every day I guess you are kind of reminded that you have this inside your body every day.” (30)* |
| Reduced cost | 10 (33.3) | *Both of the medicines have a co-pay, but they require you to have primary insurance. … often your costs can go up hundreds and hundreds of hundreds of dollars per month and that is not something that I … think I could do… spending most of your money on healthcare … can be tricky” (31)* |
| More support | 8 (26.7) | *“Actual support, you know, people that actually -- I know people -- some people do have that and, you know, if support was there it’ll be a lot easier for certain people or for a lot of people to, how do you say, be okay with it. So support would be one thing.” (11)* |
| Smaller pill size | 2 (6.7) | *“I had an issue with my other pills, they were like so big, and I had a hard time swallowing them … the fact that the pill is smaller … is good” (07)* |
