# Supplementary material for: Qualitative evaluation of survey questions to assess treatment preference for daily oral or long‑acting injectable antiretroviral therapy among people living with HIV
Source: PLoS One. 2024 Dec 27;19(12):e0309588. doi: 10.1371/journal.pone.0309588 (PMC11676866; doi:10.1371/journal.pone.0309588)
Supplement: S2 Table — (DOCX) [file pone.0309588.s002.docx]

| **Supplementary Table S2:** Participant quotes related to responses selected for long-acting injectable HIV medication or daily oral HIV medication | |
| --- | --- |
| **Response option** | **Participant quotes (Participant ID)** |
| *Long-acting, injectable ART* | |
| I was tired of taking tablet(s) for my HIV every day | *“I think that does apply to me, and that would be one of my choices. I don’t want to take a tablet every day. I would prefer to take it every so often once or twice a week, biweekly, or even monthly.” (29)* |
| It is more convenient for me to receive injections every 2 months | *“Absolutely, I agree that would be pretty convenient. It would remove a step for me at night and also I – if I were to have anxiety about missing, it would eliminate that completely. So, I think that’s positive.” (26)* |
| I do not have to worry as much about remembering to take my HIV medication every day | *“That would be my choice because like doing the injections is more likely for me to remember than to taking pills because if I’m like busy throughout the day, I wouldn’t even think about the pills, so I probably will forget, you know, unless I had like a timer or something to remind me or a daily reminder.” (07)* |
| I do not have to carry my HIV medication with me | *“I think the last question, I do not have to worry or have to carry my HIV medication with me. I think that would be an issue for me or something I’d be interested in.” (23)* |
| I have difficulty swallowing oral HIV medication | *“I have difficulty swallowing for HIV medicines, yeah, because they are big pills.” (24)* |
| I do not have to worry about others seeing or finding my HIV pills | *“I do not have to worry about others seeing or finding, yes, it’s very important. Like I say, people are very nosey, people tend to pry in your business.” (27)* |
| I do not have to think about my HIV status every day | *“Yeah, I would – it would probably be out of sight, out of mind, I believe. Yeah. I think that would change it. And yeah, I think I’d like that better.” (34)* |
| I feel more in control of managing my HIV | *“I think if it was like just a shot every two months, I would think I am more in control because now I don’t have to worry about it every day. I know it’s there, I know it’s keeping me stable. I would think I am in more control because now I can live my life more than worrying about it every day.” (27)* |
| I like more frequent interaction with my HIV provider | *“For sure because I always -- every time I go in I ask my doctor about recent findings and are there new discoveries, and then of course, I ask about my specific case or my specific situation, like how am I responding to the medication. So, I have a fairly transparent and open relationship with my provider, so I think that that would be a reason for me.” (32)* |
| I believe injections are more reliable than daily oral medication to keep my viral load undetectable | *“And then I guess also the finding injections are more reliable, that one kind of, I guess, if I was in the study and that’s what I was finding that it was still maintaining me at that level, then I would also use that as a supporting statement.” (09)* |
| *Daily oral ART* | |
| I want to avoid injection pain and side effects from the injection | *“I want to avoid pain. I thought of that one, injection pain, and side effects, any side effects.” (13)* |
| I am afraid of needles and injections | “I’m afraid of needles and injections. Okay, that’s that one right there. That’s kind of that’s true.” (24) |
| It is more convenient for me to take my HIV medication every day | *“It’s more convenient for me to take it every day like that I could just get up, drink my medication, take a shower, get dressed, and just keep, you know, go on my daily routine.” (16)* |
| I worry about others finding out that I am visiting an HIV clinic every 2 months | *“I worry about others finding out that I’m visiting HIV clinic. Yes, I do.” (34)* |
| I don't have time to attend my HIV clinic appointments every 2 months | *“I would say yes to that. That could be an inconvenience depending on someone’s work schedule, work life, things like that or personal life.” (28)* |
| It is inconvenient for me to visit the HIV clinic to receive injections | *“Yeah, I would personally think for me it’s kind of inconvenient to do that. So, yeah, that’s probably little bit applicable.” (25)* |
| I feel embarrassed getting injections | *“I think that I feel embarrassed getting injections because it depends on where it goes. Like I wouldn’t want someone seeing a bandaid and knowing I got a shot. Or if someone has to see my bits to give me a shot. I wouldn’t want that.” (06)* |
| I believe daily oral medication is more reliable than injections to keep my viral load undetectable | *“That I would select just because it’s what I know.” (02)* |
| I have experienced stigmatizing attitudes and behaviors from health professionals during injection administration | *“And additionally, if the clinic people are mean to me, I would prefer my pills. So I would pick I have experienced stigmatizing attitudes and behaviors from health professionals during injection administration.” (06)* |
| I feel more in control of managing my HIV condition | *“And I will say this statement does apply because people want to feel more in control when they do things themselves, and don’t have to necessarily rely on someone else, or something else. And having oral medication gives them that opportunity to be in control.” (29)* |
