# Supplementary figures and images for: Qualitative evaluation of survey questions to assess treatment preference for daily oral or long‑acting injectable antiretroviral therapy among people living with HIV
Source: PLoS One. 2024 Dec 27;19(12):e0309588. doi: 10.1371/journal.pone.0309588 (PMC11676866; doi:10.1371/journal.pone.0309588)

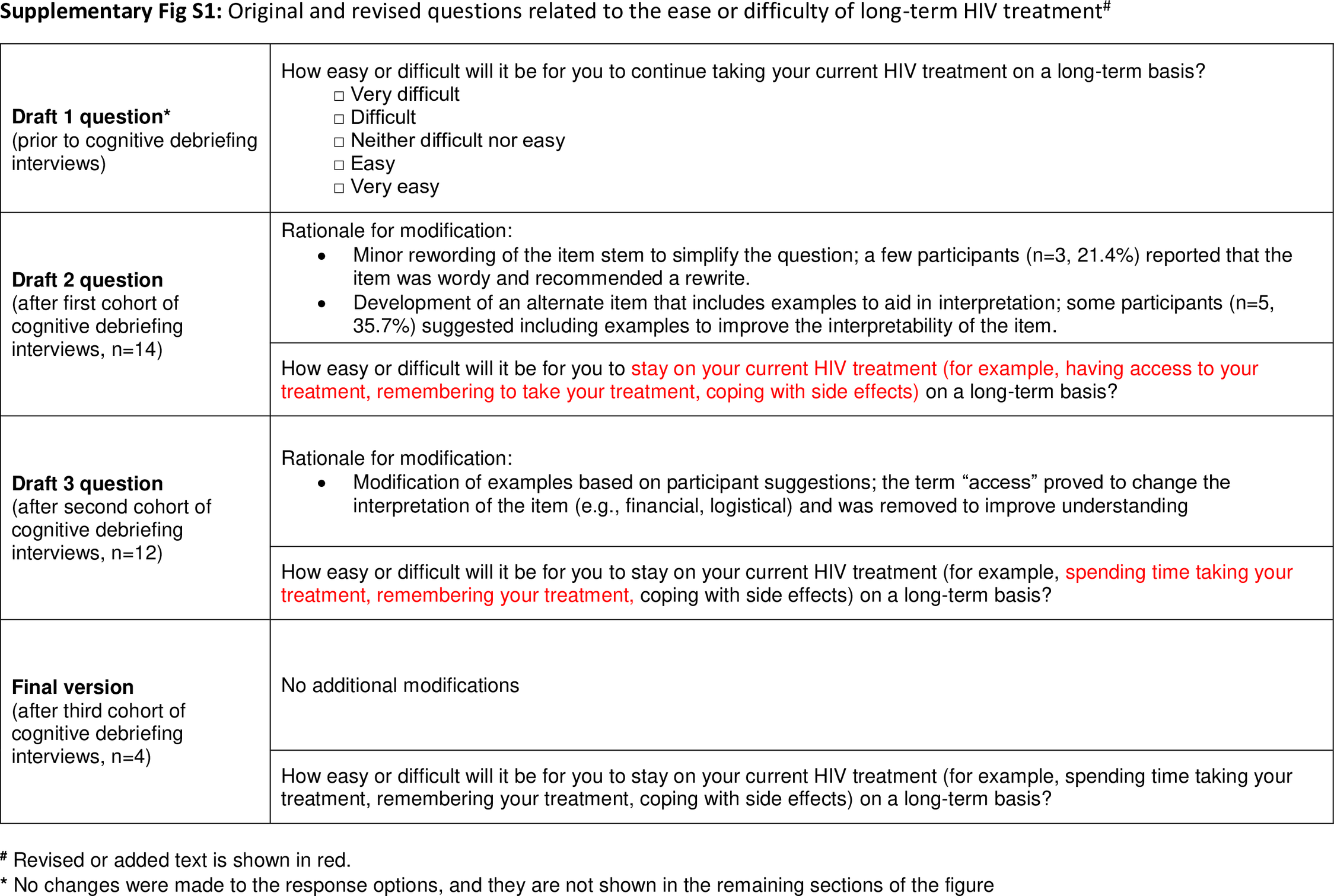

Supplement: S1 Fig — (TIF) [file pone.0309588.s003.tif]
